# Can Ecological Momentary assessments be used to investigate the person-environment interactions in people with psychosis?

**DOI:** 10.1192/j.eurpsy.2024.134

**Published:** 2024-08-27

**Authors:** I. Myin Germeys

**Affiliations:** Dept of Neurosciences, KU Leuven, Leuven, Belgium

## Abstract

**Abstract:**

Psychotic experiences show a dynamic pattern over time, often in interaction with the environment. In my talk, I will discuss how Ecological Momentary Assessment (EMA) or Experience Sampling Methodology can be used to assess psychotic symptoms in the flow of daily life. I will focus on the assessment of both positive and negative symptoms, where I will discuss both how we can measure such symptoms as well as what the dynamic patterns look like in everyday life. Furthermore, I will also focus on how ESM can be used to transfer psychological treatment to daily life using an app. I will discuss the INTERACT trial, a trial in people at the early stages of psychosis, where we investigated the effect of Acceptance And Commitment Therapy in Daily Life, compared to Treatment As Usual.

**Disclosure of Interest:**

None Declared

